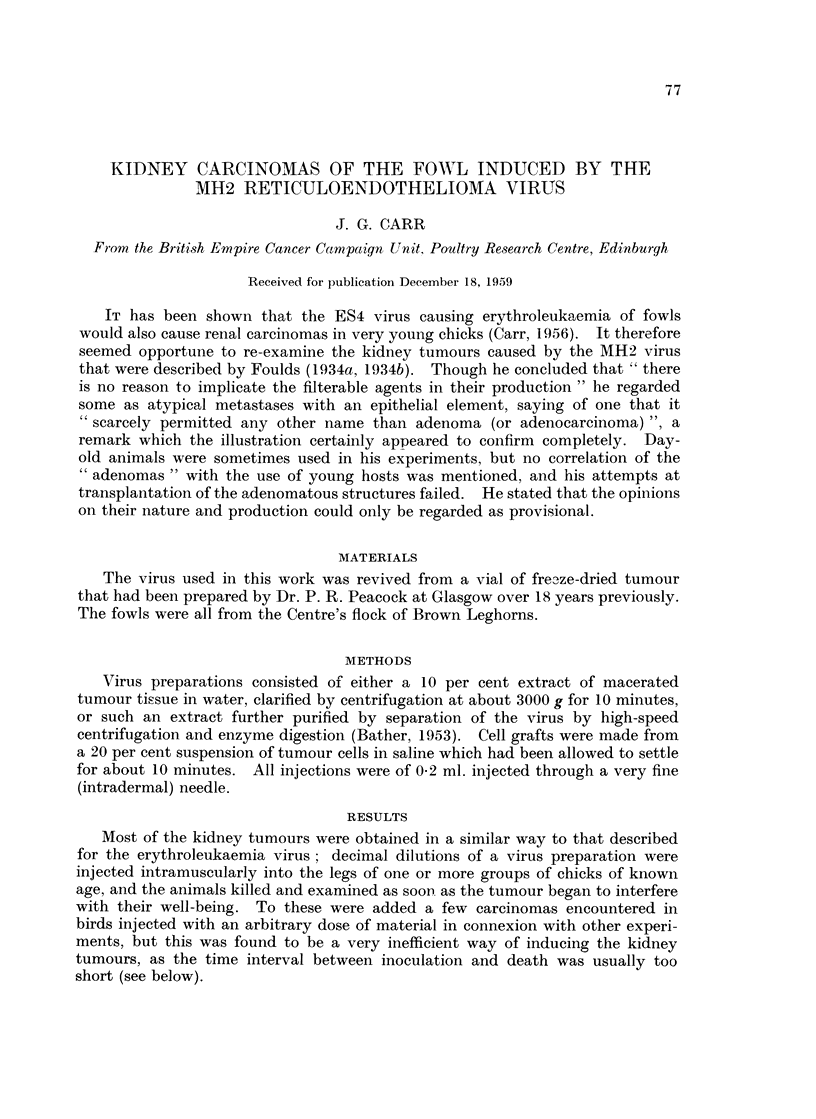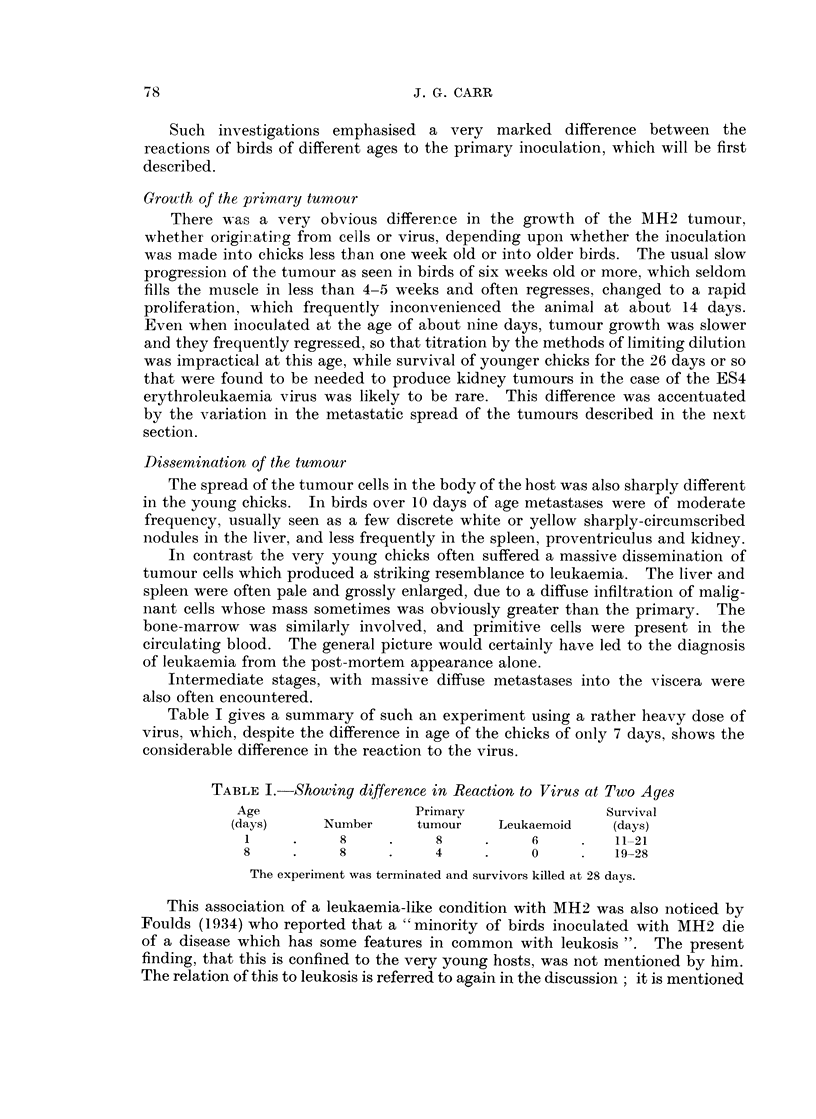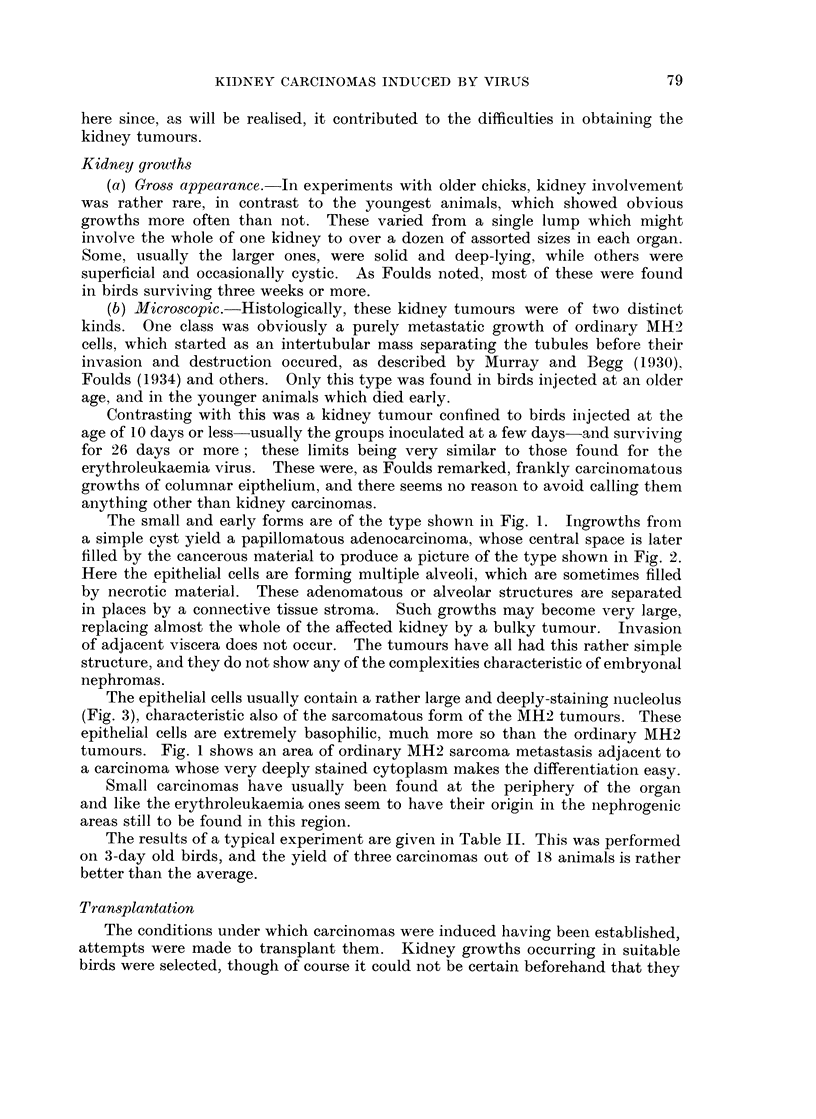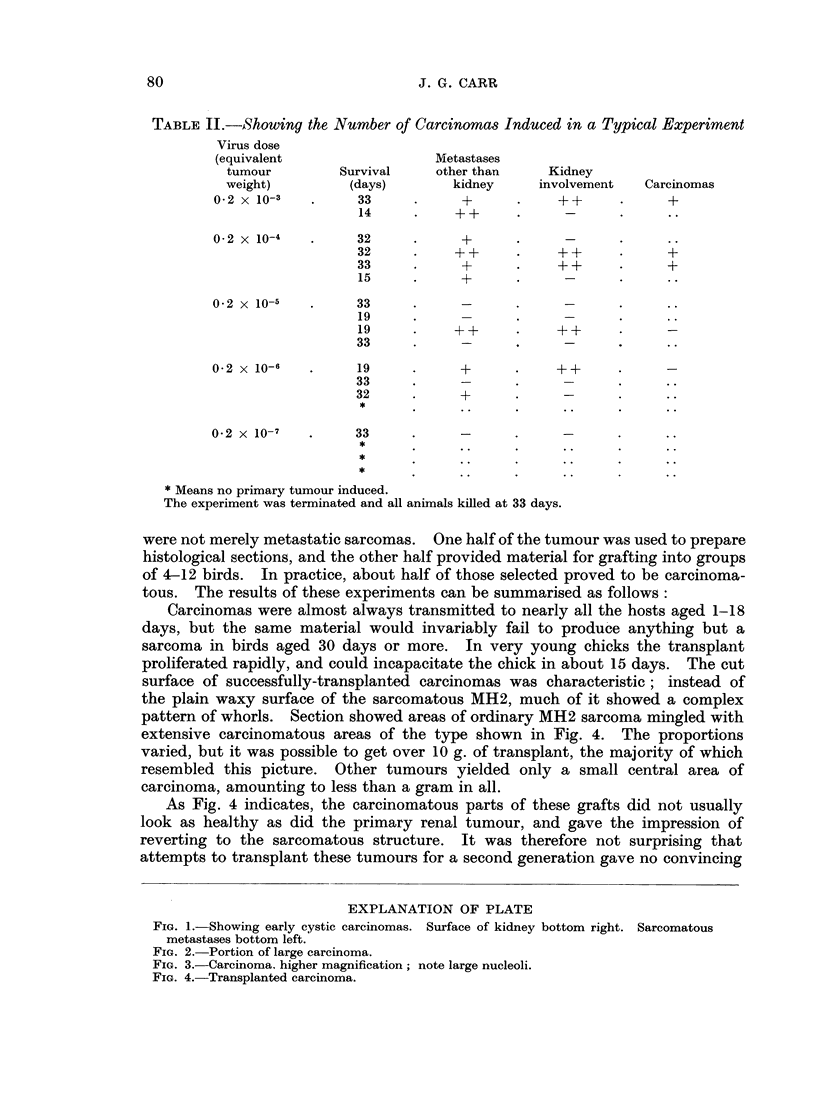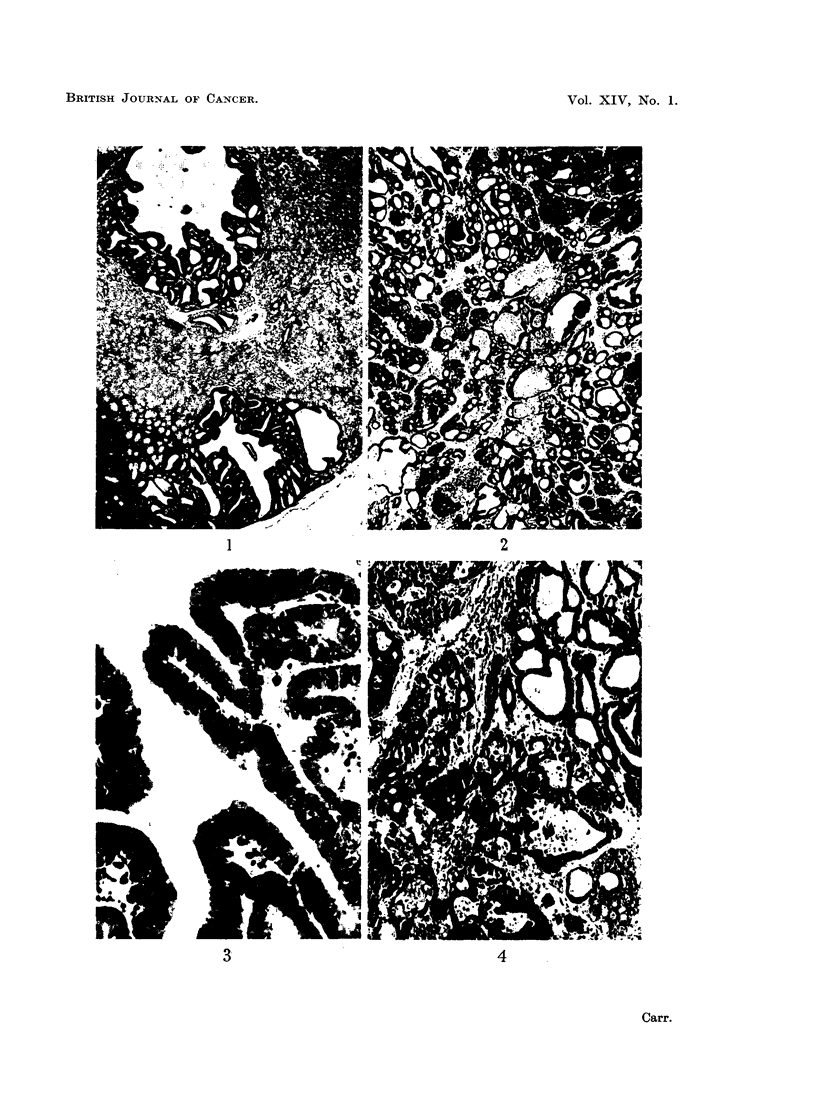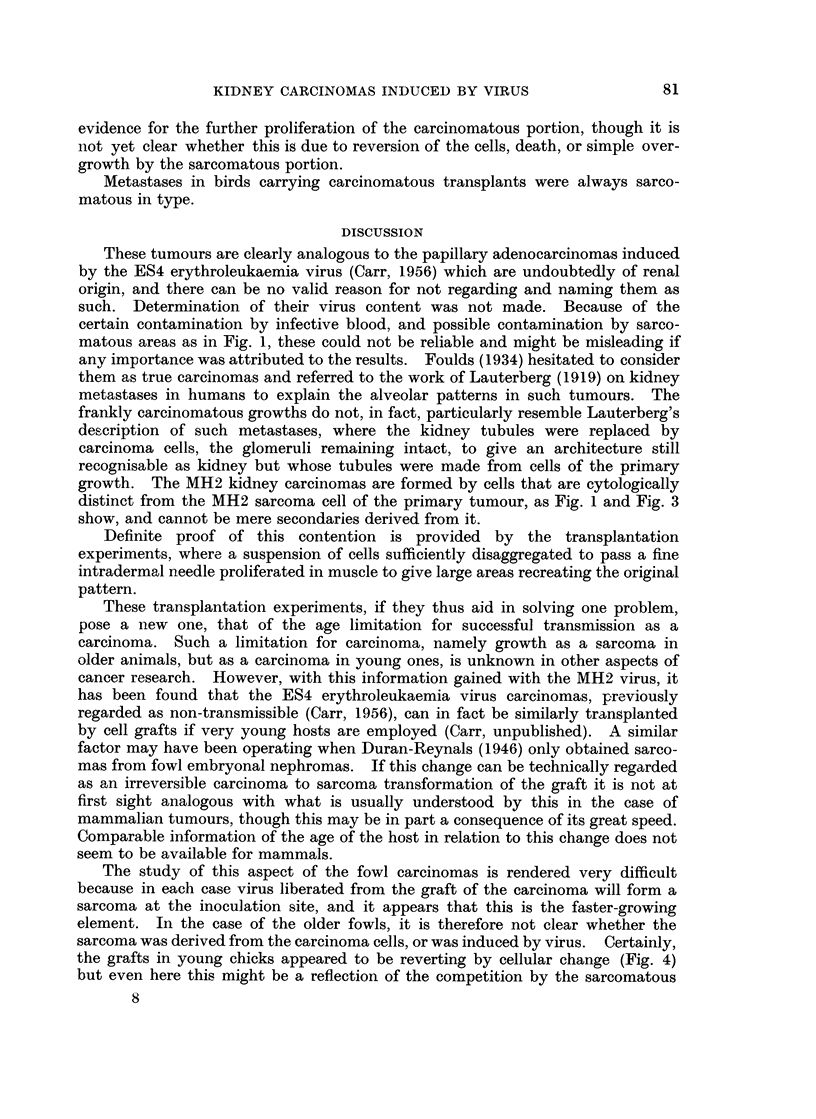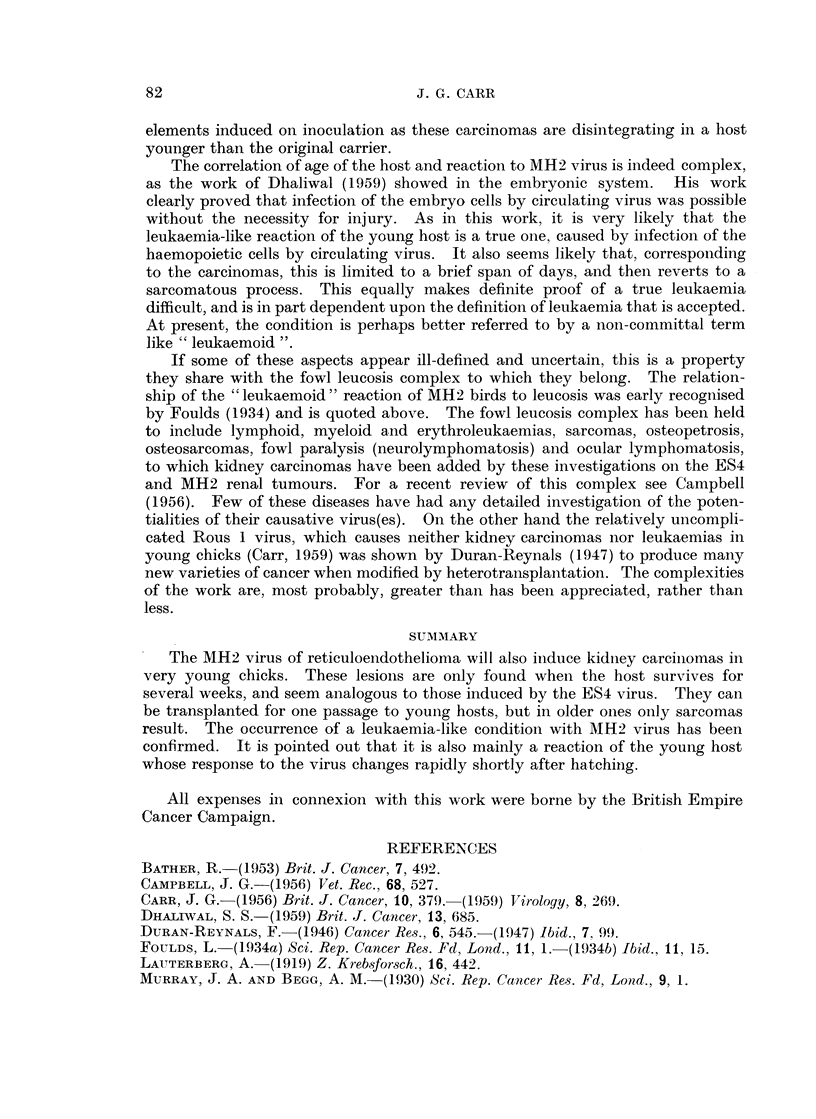# Kidney Carcinomas of the Fowl Induced by the MH2 Reticuloendothelioma Virus

**DOI:** 10.1038/bjc.1960.9

**Published:** 1960-03

**Authors:** J. G. Carr

## Abstract

**Images:**


### KIDNEY CARCINOMAS OF THE FOWL INDUCED BY THE MH2 RETICULOENDOTHELIOMA VIRUS

J. G. CARR

*From the British Empire Cancer Campaign Unit, Poultry Research Centre, Edinburgh*

Received for publication December 18, 1959

IT has been shown that the ES4 virus causing erythroleukaemia of fowls would also cause renal carcinomas in very young chicks (Carr, 1956). It therefore seemed opportune to re-examine the kidney tumours caused by the MH2 virus that were described by Foulds (1934*a*, 1934*b*). Though he concluded that "there is no reason to implicate the filterable agents in their production" he regarded some as atypical metastases with an epithelial element, saying of one that it "scarcely permitted any other name than adenoma (or adenocarcinoma)", a remark which the illustration certainly appeared to confirm completely. Day-old animals were sometimes used in his experiments, but no correlation of the "adenomas" with the use of young hosts was mentioned, and his attempts at transplantation of the adenomatous structures failed. He stated that the opinions on their nature and production could only be regarded as provisional.

### MATERIALS

The virus used in this work was revived from a vial of freeze-dried tumour that had been prepared by Dr. P. R. Peacock at Glasgow over 18 years previously. The fowls were all from the Centre's flock of Brown Leghorns.

### METHODS

Virus preparations consisted of either a 10 per cent extract of macerated tumour tissue in water, clarified by centrifugation at about 3000 *g* for 10 minutes, or such an extract further purified by separation of the virus by high-speed centrifugation and enzyme digestion (Bather, 1953). Cell grafts were made from a 20 per cent suspension of tumour cells in saline which had been allowed to settle for about 10 minutes. All injections were of 0·2 ml. injected through a very fine (intradermal) needle.

### RESULTS

Most of the kidney tumours were obtained in a similar way to that described for the erythroleukaemia virus ; decimal dilutions of a virus preparation were injected intramuscularly into the legs of one or more groups of chicks of known age, and the animals killed and examined as soon as the tumour began to interfere with their well-being. To these were added a few carcinomas encountered in birds injected with an arbitrary dose of material in connexion with other experiments, but this was found to be a very inefficient way of inducing the kidney tumours, as the time interval between inoculation and death was usually too short (see below).

Such investigations emphasised a very marked difference between the reactions of birds of different ages to the primary inoculation, which will be first described.

*Growth of the primary tumour*

There was a very obvious difference in the growth of the MH2 tumour, whether originating from cells or virus, depending upon whether the inoculation was made into chicks less than one week old or into older birds. The usual slow progression of the tumour as seen in birds of six weeks old or more, which seldom fills the muscle in less than 4–5 weeks and often regresses, changed to a rapid proliferation, which frequently inconvenienced the animal at about 14 days. Even when inoculated at the age of about nine days, tumour growth was slower and they frequently regressed, so that titration by the methods of limiting dilution was impractical at this age, while survival of younger chicks for the 26 days or so that were found to be needed to produce kidney tumours in the case of the ES4 erythroleukaemia virus was likely to be rare. This difference was accentuated by the variation in the metastatic spread of the tumours described in the next section.

*Dissemination of the tumour*

The spread of the tumour cells in the body of the host was also sharply different in the young chicks. In birds over 10 days of age metastases were of moderate frequency, usually seen as a few discrete white or yellow sharply-circumscribed nodules in the liver, and less frequently in the spleen, proventriculus and kidney.

In contrast the very young chicks often suffered a massive dissemination of tumour cells which produced a striking resemblance to leukaemia. The liver and spleen were often pale and grossly enlarged, due to a diffuse infiltration of malignant cells whose mass sometimes was obviously greater than the primary. The bone-marrow was similarly involved, and primitive cells were present in the circulating blood. The general picture would certainly have led to the diagnosis of leukaemia from the post-mortem appearance alone.

Intermediate stages, with massive diffuse metastases into the viscera were also often encountered.

Table I gives a summary of such an experiment using a rather heavy dose of virus, which, despite the difference in age of the chicks of only 7 days, shows the considerable difference in the reaction to the virus.

TABLE I.—*Showing difference in Reaction to Virus at Two Ages*

| Age (days) | Number | Primary tumour | Leukaemoid | Survival (days) |
|---|---|---|---|---|
| 1 | 8 | 8 | 6 | 11–21 |
| 8 | 8 | 4 | 0 | 19–28 |

The experiment was terminated and survivors killed at 28 days.

This association of a leukaemia-like condition with MH2 was also noticed by Foulds (1934) who reported that a "minority of birds inoculated with MH2 die of a disease which has some features in common with leukosis ". The present finding, that this is confined to the very young hosts, was not mentioned by him. The relation of this to leukosis is referred to again in the discussion ; it is mentioned here since, as will be realised, it contributed to the difficulties in obtaining the kidney tumours.

*Kidney growths*

(*a*) *Gross appearance.*—In experiments with older chicks, kidney involvement was rather rare, in contrast to the youngest animals, which showed obvious growths more often than not. These varied from a single lump which might involve the whole of one kidney to over a dozen of assorted sizes in each organ. Some, usually the larger ones, were solid and deep-lying, while others were superficial and occasionally cystic. As Foulds noted, most of these were found in birds surviving three weeks or more.

(*b*) *Microscopic.*—Histologically, these kidney tumours were of two distinct kinds. One class was obviously a purely metastatic growth of ordinary MH2 cells, which started as an intertubular mass separating the tubules before their invasion and destruction occured, as described by Murray and Begg (1930), Foulds (1934) and others. Only this type was found in birds injected at an older age, and in the younger animals which died early.

Contrasting with this was a kidney tumour confined to birds injected at the age of 10 days or less—usually the groups inoculated at a few days—and surviving for 26 days or more ; these limits being very similar to those found for the erythroleukaemia virus. These were, as Foulds remarked, frankly carcinomatous growths of columnar eipthelium, and there seems no reason to avoid calling them anything other than kidney carcinomas.

The small and early forms are of the type shown in Fig. 1. Ingrowths from a simple cyst yield a papillomatous adenocarcinoma, whose central space is later filled by the cancerous material to produce a picture of the type shown in Fig. 2. Here the epithelial cells are forming multiple alveoli, which are sometimes filled by necrotic material. These adenomatous or alveolar structures are separated in places by a connective tissue stroma. Such growths may become very large, replacing almost the whole of the affected kidney by a bulky tumour. Invasion of adjacent viscera does not occur. The tumours have all had this rather simple structure, and they do not show any of the complexities characteristic of embryonal nephromas.

The epithelial cells usually contain a rather large and deeply-staining nucleolus (Fig. 3), characteristic also of the sarcomatous form of the MH2 tumours. These epithelial cells are extremely basophilic, much more so than the ordinary MH2 tumours. Fig. 1 shows an area of ordinary MH2 sarcoma metastasis adjacent to a carcinoma whose very deeply stained cytoplasm makes the differentiation easy.

Small carcinomas have usually been found at the periphery of the organ and like the erythroleukaemia ones seem to have their origin in the nephrogenic areas still to be found in this region.

The results of a typical experiment are given in Table II. This was performed on 3-day old birds, and the yield of three carcinomas out of 18 animals is rather better than the average.

*Transplantation*

The conditions under which carcinomas were induced having been established, attempts were made to transplant them. Kidney growths occurring in suitable birds were selected, though of course it could not be certain beforehand that they

TABLE II.—*Showing the Number of Carcinomas Induced in a Typical Experiment*

| Virus dose (equivalent tumour weight) | Survival (days) | Metastases other than kidney | Kidney involvement | Carcinomas |
|---|---|---|---|---|
| $0 \cdot 2 \times 10^{-3}$ | 33 | + | ++ | + |
| | 14 | ++ | − | .. |
| $0 \cdot 2 \times 10^{-4}$ | 32 | + | − | .. |
| | 32 | ++ | ++ | + |
| | 33 | + | ++ | + |
| | 15 | + | − | .. |
| $0 \cdot 2 \times 10^{-5}$ | 33 | − | − | .. |
| | 19 | − | − | .. |
| | 19 | ++ | ++ | − |
| | 33 | − | − | .. |
| $0 \cdot 2 \times 10^{-6}$ | 19 | + | ++ | − |
| | 33 | − | − | .. |
| | 32 | + | − | .. |
| | * | .. | .. | .. |
| $0 \cdot 2 \times 10^{-7}$ | 33 | − | − | .. |
| | * | .. | .. | .. |
| | * | .. | .. | .. |
| | * | .. | .. | .. |

* Means no primary tumour induced.
The experiment was terminated and all animals killed at 33 days.

were not merely metastatic sarcomas. One half of the tumour was used to prepare histological sections, and the other half provided material for grafting into groups of 4–12 birds. In practice, about half of those selected proved to be carcinomatous. The results of these experiments can be summarised as follows :

Carcinomas were almost always transmitted to nearly all the hosts aged 1–18 days, but the same material would invariably fail to produce anything but a sarcoma in birds aged 30 days or more. In very young chicks the transplant proliferated rapidly, and could incapacitate the chick in about 15 days. The cut surface of successfully-transplanted carcinomas was characteristic ; instead of the plain waxy surface of the sarcomatous MH2, much of it showed a complex pattern of whorls. Section showed areas of ordinary MH2 sarcoma mingled with extensive carcinomatous areas of the type shown in Fig. 4. The proportions varied, but it was possible to get over 10 g. of transplant, the majority of which resembled this picture. Other tumours yielded only a small central area of carcinoma, amounting to less than a gram in all.

As Fig. 4 indicates, the carcinomatous parts of these grafts did not usually look as healthy as did the primary renal tumour, and gave the impression of reverting to the sarcomatous structure. It was therefore not surprising that attempts to transplant these tumours for a second generation gave no convincing

---

EXPLANATION OF PLATE

FIG. 1.—Showing early cystic carcinomas. Surface of kidney bottom right. Sarcomatous metastases bottom left.
FIG. 2.—Portion of large carcinoma.
FIG. 3.—Carcinoma, higher magnification ; note large nucleoli.
FIG. 4.—Transplanted carcinoma.

1

2

3

4

Carr.

evidence for the further proliferation of the carcinomatous portion, though it is not yet clear whether this is due to reversion of the cells, death, or simple overgrowth by the sarcomatous portion.

Metastases in birds carrying carcinomatous transplants were always sarcomatous in type.

### DISCUSSION

These tumours are clearly analogous to the papillary adenocarcinomas induced by the ES4 erythroleukaemia virus (Carr, 1956) which are undoubtedly of renal origin, and there can be no valid reason for not regarding and naming them as such. Determination of their virus content was not made. Because of the certain contamination by infective blood, and possible contamination by sarcomatous areas as in Fig. 1, these could not be reliable and might be misleading if any importance was attributed to the results. Foulds (1934) hesitated to consider them as true carcinomas and referred to the work of Lauterberg (1919) on kidney metastases in humans to explain the alveolar patterns in such tumours. The frankly carcinomatous growths do not, in fact, particularly resemble Lauterberg's description of such metastases, where the kidney tubules were replaced by carcinoma cells, the glomeruli remaining intact, to give an architecture still recognisable as kidney but whose tubules were made from cells of the primary growth. The MH2 kidney carcinomas are formed by cells that are cytologically distinct from the MH2 sarcoma cell of the primary tumour, as Fig. 1 and Fig. 3 show, and cannot be mere secondaries derived from it.

Definite proof of this contention is provided by the transplantation experiments, where a suspension of cells sufficiently disaggregated to pass a fine intradermal needle proliferated in muscle to give large areas recreating the original pattern.

These transplantation experiments, if they thus aid in solving one problem, pose a new one, that of the age limitation for successful transmission as a carcinoma. Such a limitation for carcinoma, namely growth as a sarcoma in older animals, but as a carcinoma in young ones, is unknown in other aspects of cancer research. However, with this information gained with the MH2 virus, it has been found that the ES4 erythroleukaemia virus carcinomas, previously regarded as non-transmissible (Carr, 1956), can in fact be similarly transplanted by cell grafts if very young hosts are employed (Carr, unpublished). A similar factor may have been operating when Duran-Reynals (1946) only obtained sarcomas from fowl embryonal nephromas. If this change can be technically regarded as an irreversible carcinoma to sarcoma transformation of the graft it is not at first sight analogous with what is usually understood by this in the case of mammalian tumours, though this may be in part a consequence of its great speed. Comparable information of the age of the host in relation to this change does not seem to be available for mammals.

The study of this aspect of the fowl carcinomas is rendered very difficult because in each case virus liberated from the graft of the carcinoma will form a sarcoma at the inoculation site, and it appears that this is the faster-growing element. In the case of the older fowls, it is therefore not clear whether the sarcoma was derived from the carcinoma cells, or was induced by virus. Certainly, the grafts in young chicks appeared to be reverting by cellular change (Fig. 4) but even here this might be a reflection of the competition by the sarcomatous elements induced on inoculation as these carcinomas are disintegrating in a host younger than the original carrier.

The correlation of age of the host and reaction to MH2 virus is indeed complex, as the work of Dhaliwal (1959) showed in the embryonic system. His work clearly proved that infection of the embryo cells by circulating virus was possible without the necessity for injury. As in this work, it is very likely that the leukaemia-like reaction of the young host is a true one, caused by infection of the haemopoietic cells by circulating virus. It also seems likely that, corresponding to the carcinomas, this is limited to a brief span of days, and then reverts to a sarcomatous process. This equally makes definite proof of a true leukaemia difficult, and is in part dependent upon the definition of leukaemia that is accepted. At present, the condition is perhaps better referred to by a non-committal term like " leukaemoid ".

If some of these aspects appear ill-defined and uncertain, this is a property they share with the fowl leucosis complex to which they belong. The relationship of the "leukaemoid" reaction of MH2 birds to leucosis was early recognised by Foulds (1934) and is quoted above. The fowl leucosis complex has been held to include lymphoid, myeloid and erythroleukaemias, sarcomas, osteopetrosis, osteosarcomas, fowl paralysis (neurolymphomatosis) and ocular lymphomatosis, to which kidney carcinomas have been added by these investigations on the ES4 and MH2 renal tumours. For a recent review of this complex see Campbell (1956). Few of these diseases have had any detailed investigation of the potentialities of their causative virus(es). On the other hand the relatively uncomplicated Rous 1 virus, which causes neither kidney carcinomas nor leukaemias in young chicks (Carr, 1959) was shown by Duran-Reynals (1947) to produce many new varieties of cancer when modified by heterotransplantation. The complexities of the work are, most probably, greater than has been appreciated, rather than less.

### SUMMARY

The MH2 virus of reticuloendothelioma will also induce kidney carcinomas in very young chicks. These lesions are only found when the host survives for several weeks, and seem analogous to those induced by the ES4 virus. They can be transplanted for one passage to young hosts, but in older ones only sarcomas result. The occurrence of a leukaemia-like condition with MH2 virus has been confirmed. It is pointed out that it is also mainly a reaction of the young host whose response to the virus changes rapidly shortly after hatching.

All expenses in connexion with this work were borne by the British Empire Cancer Campaign.

### REFERENCES

Bather, R.—(1953) *Brit. J. Cancer*, **7**, 492.
Campbell, J. G.—(1956) *Vet. Rec.*, **68**, 527.
Carr, J. G.—(1956) *Brit. J. Cancer*, **10**, 379.—(1959) *Virology*, **8**, 269.
Dhaliwal, S. S.—(1959) *Brit. J. Cancer*, **13**, 685.
Duran-Reynals, F.—(1946) *Cancer Res.*, **6**, 545.—(1947) *Ibid.*, **7**, 99.
Foulds, L.—(1934*a*) *Sci. Rep. Cancer Res. Fd, Lond.*, **11**, 1.—(1934*b*) *Ibid.*, **11**, 15.
Lauterberg, A.—(1919) *Z. Krebsforsch.*, **16**, 442.
Murray, J. A. and Begg, A. M.—(1930) *Sci. Rep. Cancer Res. Fd, Lond.*, **9**, 1.